# Leiomyosarcoma of the thyroid gland: A case report and literature review

**DOI:** 10.3892/ol.2014.1853

**Published:** 2014-02-04

**Authors:** GIOVANNI CONZO, GIANCARLO CANDELA, ERNESTO TARTAGLIA, CLAUDIO GAMBARDELLA, CLAUDIO MAURIELLO, GUIDO PETTINATO, GIUSEPPE BELLASTELLA, KATHRINE ESPOSITO, LUIGI SANTINI

**Affiliations:** 1 Department of Anaesthesiology, Surgical and Emergency Science VII Division of General Surgery, Second University of Naples, Naples I-80131, Italy; 2 Thoracic Surgery Unit, Second University of Naples, Naples I-80138, Italy; 3 Department of Cardio-Thoracic and Respiratory Sciences, Unit of Endocrinology and Diabetes Clinic, Second University of Naples, Naples I-80131, Italy

**Keywords:** thyroid leiomyosarcoma, smooth muscle tumor, total thyroidectomy, fine-needle aspiration biopsy

## Abstract

Primary smooth muscle tumors of the thyroid gland are extremely rare neoplasms. Due to their rarity, clinical case studies concerning management are lacking. According to a literature review, only 19 cases of primary thyroid leiomyosarcomas (TLs) have been reported. In the majority of patients, the prognosis is poor since adjuvant radiochemotherapy is ineffective on local recurrence and on long-term survival. In this study, we report the case of a 77-year-old male affected by a rapidly enlarging mass of the anterior neck, associated with bilateral lung metastases, and increasing dysphagia and dyspnea during the previous 6 months. A Tir4 neoplasm fine needle cytological diagnosis of the right thyroid lobe was reached and the patient underwent total thyroidectomy (TT). Definitive histological examination identified a TL. The patient succumbed 40 days later due to respiratory distress. A literature review was performed and TL differential diagnoses, management, including alternative treatment strategies, and adjuvant therapy were analyzed. TL is an aggressive rare mesenchymal malignant tumor. Although an improved multimodal approach is often necessary, TT and neck dissection represent the treatment of choice and are often the only possible therapy. Adjuvant radiochemotherapy appears to be ineffective and a high mortality rate is observed. TL remains a fatal tumor, and innovative and more effective therapeutic strategies to improve management and outcomes are required.

## Introduction

Primary thyroid leiomyosarcoma (TL) represents just 0.014% of primary thyroid cancers (1) and is associated with an extremely aggressive clinical course, leading to an extremely poor 5-year survival rate. According to the World Health Organization’s histological classification of thyroid and parathyroid tumors, smooth muscle tumors can be either benign (leiomyoma) or malignant (leiomyosarcoma) (2). Primary TL is a mesenchymal malignant tumor with smooth muscle differentiation, arising from the smooth muscle cells of the vessels located in the thyroid capsule (1). Pathological examination may be ineffective in distinguishing primary from metastatic TL and clinical examination and instrumental diagnostic work-up are required. Soft tissues, gastrointestinal tract and particularly pelvic organs represent the most common sites of origin (3). Cytological evaluation reveals spindle cells, which may also be present in other more common primary tumors of the thyroid gland, such as medullary or anaplastic thyroid cancer (4). Therefore, as in the reported case, preoperative fine needle aspiration biopsy (FNAB) diagnosis can be extremely hard. According to our knowledge, only 19 cases of TL, not responding to any therapeutic approach, associated with a dismal prognosis have been described (3). We present cytohistopathological patterns and the clinical course of a patient affected by TL. A literature analysis was performed using a PubMed data base search, using keywords thyroid leiomyosarcoma and thyroid smooth cell tumor. Incidence, diagnostic work-up, management and most recent drug protocols were evaluated in order to provide the latest results about this issue. Written informed consent was obtained from the patient.

## Case report

### Case presentation and diagnosis

**Figure 1:**
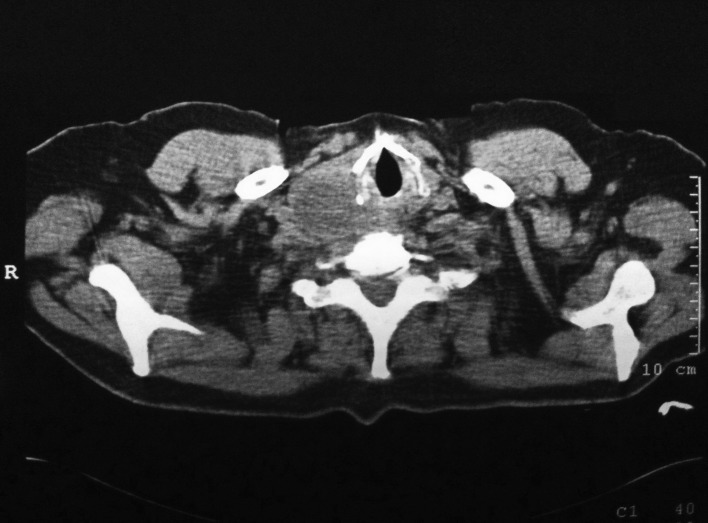
Computed tomography scan depicting a nodular lesion of the right lobe of the thyroid gland.

**Figure 2:**
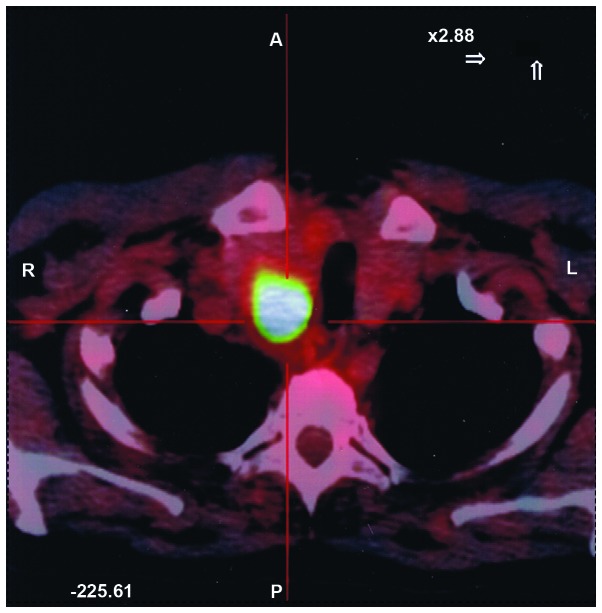
Positron emission tomography scan showing heterogeneous uptake in the right lobe of the thyroid gland (SUV 10.6).

In June 2012, a 77-year-old male was admitted to the Department of Anaesthesiology, Surgical and Emergency Science VII Division of General Surgery, Second University of Naples (Naples, Italy) with a clinical history of a recently arisen neck mass, resulting in dysphagia and dyspnea. Thyroid-stimulating hormone, calcitonin, thyroglobulin and carcinoembryonic antigen levels were in the normal range. Ultrasound examination revealed a hypoechoic nodular lesion of the right lobe of the thyroid gland, with irregular margins and a central cystic area (39.6×35.3×34.1 mm) confirmed by a contrast medium computed tomography (CT) scan (Fig. 1); bilateral multiple pulmonary lesions were also identified. A positron emission tomography scan showed a heterogeneous uptake in the right lobe of the thyroid (SUV 10.6) and in the two pulmonary fields (SUV 5.0–8.0) (Fig. 2). A FNAB revealed isolated and clustered spindle cells with an epithelioid aspect.

### Surgery and follow-up

The postoperative course following a total thyroidectomy (TT) was uneventful and the patient was discharged on day 3. Adjuvant postoperative therapy was not performed due to the poor general clinical conditions. The patient succumbed 40 days after surgery, due to respiratory distress.

### Cytopathological and histopathological findings

FNAB was performed using a 25-mm/23G needle connected to a 10-ml plastic syringe connected to a Cameco holder. Several slides were obtained, which were either wet-fixed in 95% ethanol or air-dried and stained, respectively, with Papanicolaou, May-Grumwald Giemsa and alkaline Congo Red stains.

Cytological smears showed plump spindle cells with elongated, blunt-ended nuclei and acidophilic, fibrillary cytoplasm; these cells were isolated or in clusters, in a proteinaceous and necrotic background. There were also isolated cells with abundant eosinophilic cytoplasm and epithelioid features. These features were highly suspected for a malignancy of mesenchymal origin.

Grossly, the tumor originated within the right lobe of the thyroid gland and measured 4.5–6.5 cm in the greatest diameter. The mass was mainly solid, with areas of fresh tumor necrosis, hemorrhage and cystic degeneration, and was not well-circumscribed.

The histological pattern of growth was predominantly solid, with clusters of epithelioid cells mixed with areas of spindle-shaped and pleomorphic cells. These clusters were interspersed with areas of marked sclerosis and with large areas of coagulative necrosis and hemorrhage. The single cells showed considerable variation in nuclear size, shape and morphology; however, the majority of neoplastic cells presented hyperchromatic nuclei and abundant eosinophilic cytoplasm that showed focal, irregular, intracytoplasmic vacuoles. A distinct fibrillarity was present within the cytoplasm of a number of cells. The mitotic rate was extremely high (25 mitosis/10 high-power field), and atypical mitotic figures were also present. The neoplasia showed invasion of the periglandular fat tissue.

Immunohistochemical staining showed diffused and marked reactivity with vimentin and H-caldesmon, and focally, with smooth muscle actin and specific muscle actin. No reactivity was shown for all keratins tested (pan-cytokeratin AE1-AE3, CK7, CK19, CK5/6 and CK8/18), EMA, TTF-1, thyroglobulin and for the endothelial markers, CD31 and factor VIII.

Morphological and immunophenotypic features were suggestive of a malignant neoplasia with mesenchymal origin, such as a primary leiomyosarcoma of the thyroid gland.

## Discussion

Papillary and follicular variants are the most frequent thyroid neoplasms, followed by medullary cancers, often part of multiple endocrine neoplasia type 2 (5–8). Extremely rare and aggressive, anaplastic carcinoma, considered a fatal tumor, is associated with a poor survival rate, as reported for sarcomatoid carcinoma of other origins (9). In the last 10 years, ultrasonography-guided FNAB has allowed a more precocious diagnosis (10,11). Primary TL, an extremely rare neoplasm, may originate from smooth muscle cells of the capsula vessels. Metaplasia from a previously existing thyroid anaplastic carcinoma should be considered (1,12,13). At the time of first diagnosis, TL is frequently associated with distant metastases. It is a fatal tumor with a 1-year survival rate of <20%. Grossly, TL are large fleshy white-gray masses, with foci of fresh tumor necrosis and hemorrhage, and a tendency for cystic degeneration. Microscopically, the pattern of growth is usually fascicular, with tumor bundles intersecting each other. Certain tumors also present areas with a whorled appearance. The individual neoplastic cells are elongated, with abundant acidophilic fibrillary cytoplasm; the nucleus is generally centrally located and typically blunt-ended or ‘cigar-shaped’. These features also appear on cytological samples. The degree of nuclear atypia is highly variable and the mitotic activity varies considerably. High mitotic activity is virtually diagnostic of malignancy, although a TL must be strongly suspected for a tumor that is widely necrotic, hemorrhagic and with significant atypia, even if the mitotic index is low.

Immunohistochemically, TL show reactivity for vimentin, smooth muscle actin, muscle-specific actin, smooth muscle myosin, desmin, H-caldesmon and basal lamina components, including laminin and type IV collagen. H-caldesmon is a muscle marker used to discriminate between smooth muscle cells and myofibroblasts; this marker appears to be associated with the degree of differentiation. Other antigens sporadically identified in TL are S-100 protein, estrogen and progesterone receptor proteins, raising the possibility of hormonal responsiveness (14).

In our referral endocrine surgery center, 250–300 thyroidectomies are performed per year. One case of primary TL has been observed in the last 33 years. To our knowledge, only 19 cases have been described so far in the international literature, making the present case the twentieth reported case of primary TL (3). In the majority of cases, patients are generally female in their sixth and seventh decades (15) and complaining of local compressive symptoms, in addition to neck pain and tenderness. Differential diagnosis includes anaplastic or medullary thyroid carcinoma, solitary fibrous tumor and spindle epithelial tumor with thymus-like differentiation (SETTLE), due to the presence in each variant of spindle cell elements.

The majority of anaplastic (undifferentiated) thyroid tumors show ‘sarcoma-like’ features, with spindle-shaped neoplastic cells arranged in a fascicular or whorled pattern of growth. Immunohistochemical stains for keratins, expressed in 50–100% of cases, confirm the epithelial nature of the tumor. Medullary thyroid carcinoma cells may be spindle-shaped; however, immunohistochemically, they are reactive for keratins, thyroid transcription factor-1 (thyroglobulin-negative), neuron-specific enolase, chromogranin (A, B and C), synaptophysin, opioid peptides and calcitonin. SETTLE, occurring in children and adolescents, is a rare tumor usually located in the thyroid gland and perithyroid tissue. Histologically, it is a biphasic neoplasm composed of spindle cells admixed with epithelial structures, generally without atypia.

TL preoperative diagnosis can be extremely difficult. It is important to discriminate between primary TL and ‘non-thyroid’ cervical leiomyosarcoma (1% of head and neck sarcoma) and, furthermore, exclude a metastatic origin from stomach, pelvis and soft tissue (16). CT scan and magnetic resonance imaging are useful for defining the local extent of disease and for identifying distant metastases. To date, it is not clear whether therapy is effective in prolonging survival, as demonstrated in 19 reported cases (3). Rapid locoregional infiltration and diffuse brain or lung metastases are responsible for the high mortality rate. Total or near-total thyroidectomy, for the majority of thyroid pathologies, associated with therapeutic modified radical neck dissection should be considered for intrathyroidal disease (17–21). Chemotherapy has not shown any therapeutic efficacy. Wang *et al* and Raspollini *et al* reported interesting data in the management of thyroid and uterus leiomyosarcomas through the overexpression of c-Kit proto-oncogene, a tyrosine kinase receptor (4,22). However, the use of imatinib mesylate (tyrosine kinase inhibitor) did not prevent the relapse and the fatal outcome in one patient with TL associated with lung metastases (23). In the case of locoregional infiltrating disease, surgery may be performed to prevent airway or esophageal obstruction. Often, therapies do not produce any clinical benefit, only palliative results.

TL remains a fatal tumor, invariably associated with a dismal prognosis, and, although notable improvements in oncology, an efficacious multimodal treatment protocol is lacking. To modify the poor surgical outcomes, novel and effective adjuvant therapeutic strategies, based on a molecular approach, are required.
